# Neonatal encephalopathy: a diffusional kurtosis imaging analysis of white matter to assess injury severity and recovery

**DOI:** 10.1038/s41390-025-04434-x

**Published:** 2025-10-30

**Authors:** Hunter G. Moss, Milad Yazdani, Jens H. Jensen, Lakshmi Katikaneni, Donald B. Wiest, Carol L. Wagner, Dorothea D. Jenkins

**Affiliations:** 1 Department of Neuroscience, Medical University of South Carolina, Charleston, SC USA; 2 Center for Biomedical Imaging, Medical University of South Carolina, Charleston, SC USA; 3 Fairfax Radiology Centers, Fairfax, VA USA; 4 Department of Radiology and Radiological Science, Medical University of South Carolina, Charleston, SC USA; 5 Department of Pediatrics, Medical University of South Carolina, Charleston, SC USA; 6 College of Pharmacy, Medical University of South Carolina, Charleston, SC USA

## Abstract

**Background:**

Diffusional kurtosis imaging (DKI) may be more sensitive to white matter (WM) injury than diffusion tensor imaging (DTI) in infants with neonatal encephalopathy (NE) presumed from hypoxic ischemic encephalopathy (HIE). We hypothesized that DKI would differentiate encephalopathy severity, predict outcomes, and indicate treatment response.

**Methods:**

DKI was acquired using 3 T MRI in neonates with HIE on day 4–10 following hypothermia (*n* = 21, HIE-C), or day 5-6 (*n* = 16, NVD-A) and 2–6 weeks (n = 18, NVD-R) in infants who received N-acetylcysteine and vitamin D (NVD) with hypothermia. We utilized tensor-derived diffusion metrics in generalized linear models to estimate Peabody Gross Motor, Cognitive Adaptive, Clinical Linguistic, and Auditory Milestones test scores.

**Results:**

Mean kurtosis differentiated stage 3 and 2 encephalopathy in all cohorts. The recovery scan predicted outcomes better than the acute scan. Kurtosis metrics and Vitamin D dose were significant determinants of gross motor and cognitive scores in NVD-R group.

**Conclusion:**

Kurtosis reflects the degree of acute CNS injury and was sensitive to treatment effects in term infants with a clinical diagnosis of HIE. Our data are consistent with prior work in adult stroke, which indicates that diffusivity and kurtosis at different stages of recovery capture distinct aspects of WM injury.

**Impact:**

- Diffusional kurtosis imaging (DKI) is an advanced diffusion MRI modality rarely applied in pediatric imaging that quantifies the complexity of water movement in white matter (WM), and which offers increased sensitivity to the severity of injury and recovery after neonatal encephalopathy due to presumed hypoxic-ischemic brain injury.
- We demonstrate that kurtosis in early-myelinating WM tracts differentiates the severity of neonatal encephalopathy (NE) better than diffusivity, which may have therapeutic implications.
- DKI provides complementary information about WM injury and recovery after NE beyond current methods, which are lacking in subjective clinical MRI assessments.
- DKI indicates the effect of therapeutics on WM injury in NE.

## Introduction

Hypoxic ischemic injury can be aggravated by pre-existing fetal neuroinflammation from intrauterine oxidative stress or infection, resulting in poor neurodevelopmental outcomes.^1–6^ Therapeutic hypothermia (TH) for 72 h improves the outcomes of cerebral palsy (CP) and some cognitive deficits,^7–9^ but offers incomplete neuroprotection as 45-55% of infants have deficits in motor abilities, IQ, executive functioning, vision, or speech, and neuro-behavior.^7,10^ While additional treatments target early and late oxidative stress, inflammation, and apoptosis,^11–13^ we have few biomarkers for use in these clinical trials to accurately quantify the response to therapy in both gray and white matter (WM). N-acetyl aspartate or lactate MR spectroscopy (MRS) ratios in the basal ganglia (BG) are the best prognostic markers for motor outcomes such as CP,^14,15^ but are not specified in WM injury (WMI) after TH for HIE. In adult non-human primate and human stroke, WMI is progressive and is closely related to functional outcome.^16,17^ Furthermore, diffusion imaging revealed temporal differences in the evolution of derived metrics between gray and WM injury after HI. Imaging at 4–10 days post-injury is optimal for assessing BG MRS but may lead to pseudo-normalization of specific diffusion abnormalities.^17–20^

In neonatal HIE, there is a need to identify more effective diffusion MR imaging (dMRI) techniques for assessing WMI and to determine the optimal time for WMI assessment. Neonatal MR brain imaging for HIE routinely includes dMRI to highlight ischemic regions,^21^ but only qualitatively and with poor inter-reader reliability in the clinical setting.^22^ Measuring the dMRI signal in multiple directions enables more quantitative dMRI analysis methods to approximate the actual diffusion probability distribution function. Diffusion tensor imaging (DTI)^23^ is one such analysis that typically gathers 12-30 diffusion encoding directions for more accurate estimation of the diffusion tensor. DTI has been widely applied in clinical research, utilizing pure diffusion metrics such as the mean diffusivity (MD), which relates to the amplitude of diffusion, and the fractional anisotropy (FA), which reflects the degree of diffusion directionality within the tissue. DTI assumes a mono-exponential signal decay, as implied by a Gaussian probability distribution function for the water diffusion dynamics. However, non-Gaussian effects, caused by bulk water molecule interactions with underlying tissue structures, are apparent in brain tissue when the diffusion weighting (i.e., *b*-value) exceeds approximately 1000 s/mm^2^. This limitation hinders DTI’s potential to identify microstructural changes in the largely unmyelinated developing brain after injury.

Advanced quantitative diffusion neuroimaging methods, beyond DTI, may offer improved prognostic tools and facilitate the clinical translation of promising therapeutics. Diffusional kurtosis imaging (DKI),^24^ a generalization of DTI, accounts for the non-Gaussian aspect of tissue water diffusion that arises from barriers such as cell walls, intracellular cytoarchitecture, and subcellular structures. The dimensionless summary statistic, kurtosis, quantifies the amount of non-Gaussianity within the tissue, such that higher kurtosis values indicate greater obstruction to water diffusion, and therefore, increased tissue complexity. In contrast, the MD will be higher in the absence of obstructions, such as is seen in the ventricles. A DKI analysis estimates the diffusion tensor, like DTI, as well as the kurtosis tensor from which we can extract the mean kurtosis (MK), the average kurtosis over all directions, and the kurtosis fractional anisotropy (KFA), which is the amount of anisotropy in the kurtosis tensor. Where multiple axon fiber bundles cross in WM, directional variation in the measured water movement can cancel out, resulting in artificially low FA values.^25–27^ An extreme example of this would be a voxel with two orthogonal axon bundles, each individually exhibiting high anisotropy, which would be estimated to have a low FA. Although more sensitive to noise variations, the KFA does not suffer from this well-known FA “crossing fiber” artifact, such that both DKI and DTI independently highlight different aspects of anisotropy within the tissue.^28^

In adult stroke, kurtosis metrics improve predictions of axonal reorganization, WM recovery, and long-term motor outcome compared with diffusivity metrics.^16,29–31^ DKI may offer similar advantages in the developing neonatal brain, though it has not been widely studied in this population. DKI has been used to document age-related tissue microstructural changes in neonates, pre-term infants, and in healthy term-infants up to 4 years of age, and providing some normative values.^32,33^ In another report, WM tract integrity as modeled by DKI^34^ was used to identify term and preterm neonates with and without encephalopathy presumed due to HIE,^35^ although the main kurtosis parameters were not presented.

Here, we applied DKI in neonates who qualified for and received TH after a clinical diagnosis of HIE to determine which diffusion metrics would best differentiate moderate and severe NE, and whether the diffusion metrics depended on the specific WM fiber tract region-of-interest (ROI) or time of assessment after injury (i.e., acute or recovery phase). We hypothesized that kurtosis would correlate with concurrent clinical severity, predict long-term outcomes, and be sensitive to treatment effects in neonates with NE shortly after birth, making it a potentially valuable biomarker.

## Methods

### Participants and recruitment cohorts

Cohorts in this study included neonates with a clinical diagnosis of HIE receiving TH for moderate to severe encephalopathy,^36^ who had DKI datasets acquired prospectively under study protocols approved by the Medical University of South Carolina’s Institutional Review Board and conducted under the ethical principles of the Declaration of Helsinki. All enrolled infants had moderate to severe encephalopathy as indicated by modified Sarnat staging^36^ and qualified for 72 h of TH according to clinical HIE criteria: gestational age (GA) ≥ 34 weeks, one factor indicating an acute sentinel event (cord or baby pH ≤ 7.0, base deficit ≥ −13, Apgar score ≤ 5 at 10 min, or continued resuscitation after 5 min due to absence of respiratory effort), and two signs of stage 2 or 3 encephalopathy (decreased tone, activity, or reflexes; seizures; posturing/clonus).^12,37,38^ This simplified neurologic exam protocol differs nominally from Shankaran’s modified Sarnat, which stipulates 3 neurologic exam findings and counts decreased activity and lethargy as separate findings, whereas they are counted as one criteria in our protocol (decreased activity) as they are in the original Sarnat scoring.^36^ Multiple types of Sarnat scoring have been validated and associated with neurologic outcomes, including the total Sarnat score,^39^ Miller’s encephalopathy score,^40^ modified Sarnat score^41^ and our simplified Sarnat score^37,38^ and our outcomes using this protocol are the same as in other phase III studies.

**Fig. 1:**
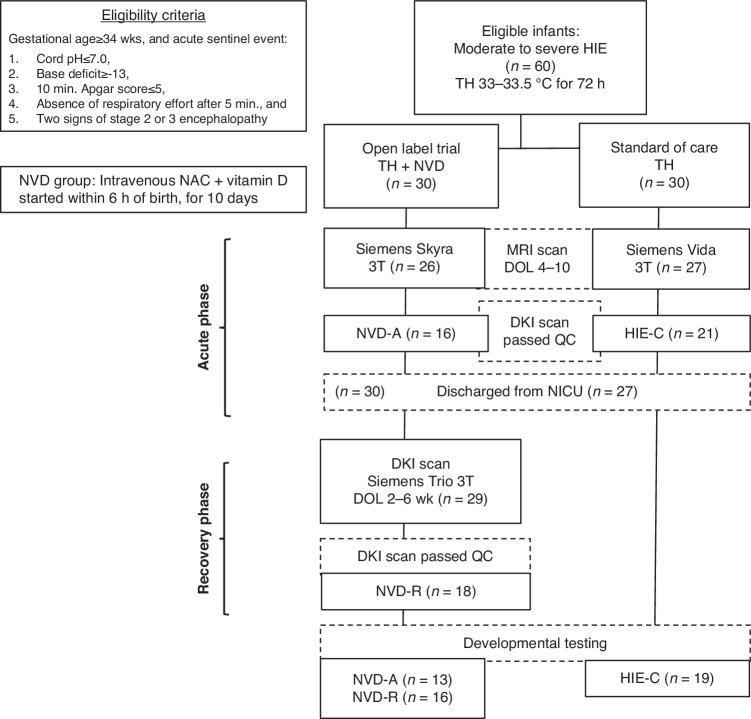
Consort diagram detailing the neonatal encephalopathic cohorts, including eligibility criteria, scanners and enrollment.

A consort diagram of the different cohorts is provided in Fig. 1 and demographics for the cohorts are given in Table 1. The first cohort was prospectively enrolled from 2014 to 2017 after parental consent to receive N-acetylcysteine and active vitamin D (NVD) in addition to TH in an open label trial in neonates with moderate to severe HIE (NCT 04643821).^12,13^ This cohort had acute DKI data gathered on day of life 4–6 (NVD acute phase: NVD-A, *n* = 16). Scans were delayed in two infants with severe hypoxia due to pulmonary hypertension requiring extra-corporeal membrane oxygenation (ECMO). The NVD cohort had a second scan in the recovery period following discharge from the NICU at 2–6 weeks (NVD recovery phase: NVD-R, *n* = 18). For the control group of HIE neonates who received TH only, after parental consent, we prospectively collected acute DKI data on day of life 5–9 from 2019 to 2020 (HIE-C, *n* = 21). Some scans were delayed from the desired 4–7 day window due to clinical instability or emergent cases. All neonates met the same inclusion criteria for cooling, and cooling protocols were uniform across cohorts.

**Table 1:** Demographics by cohort

	NVD-A			NVD-R			HIE-C		
	Stage 2 (*N* = 6)	Stage 3 (*N* = 10)	Total (*N* = 16)	Stage 2 (*N* = 7)	Stage 3 (*N* = 11)	Total (*N* = 18)	Stage 2 (*N* = 12)	Stage 3 (*N* = 9)	Total (*N* = 21)
Gender^b^									
Female	0 (0%)	3 (30%)	3 (19%)	3 (43%)	3 (27%)	6 (33%)	6 (50%)	3 (33%)	9 (43%)
Male	6 (100%)	7 (70%)	13 (81%)	4 (57%)	8 (73%)	12 (67%)	6 (50%)	6 (67%)	12 (57%)
GA Birth(wks)^a^	38.6 (1.4)	38.0 (1.5)	38.2 (1.5)	38.2 (1.7)	38.0 (1.3)	38.1 (1.4)	38.7 (1.2)	38.9 (1.3)	38.8 (1.2)
GA MRI (wks)^a^	39.4 (1.4)	39.3 (1.5)	39.3 (1.4)	41.2 (2.7)	44.2 (4.5)	43.1 (4.1)	39.6 (1.1)	39.7 (1.2)	39.7 (1.1)
Race^c^									
White	5 (31%)	5 (31%)	10 (62%)	4 (22%)	6 (33%)	10 (56%)	1 (8%)	4 (44%)	5 (24%)
Black	1 (6%)	4 (25%)	5 (31%)	3 (17%)	4 (22%)	7 (39%)	11 (92%)	5 (56%)	16 (76%)
Hispanic	0 (0%)	1 (6%)	1 (6%)	0 (0.0%)	1 (6%)	1 (6%)			
Developmental follow-up (mo)			31 (15)			34 (15)			17 (7)
MRI abnormalities									
BG infarcts			4 (25%)			5 (28%)			4 (20%)
PVWM infarcts			4 (25%)			4 (22%)			5 (24%)
ICH			4 (25%)			2 (11%)			2 (10%)
IVH, SAH, SDH			8 (50%)			6 (33%)			10 (50%)
NAC dose^b^									
25 mg/kg	5 (31%)	8 (50%)	13 (81%)	4 (22%)	8 (44%)	12 (67%)			
40 mg/kg	1 (6%)	2 (13%)	3 (19%)	3 (17%)	3 (17%)	6 (33%)			
Vitamin-D dose^b^									
0.03 mcg/kg	4 (25%)	4 (25%)	8 (50%)	5 (28%)	7 (39%)	12 (67%)			
0.05 mcg/kg	2 (12%)	6 (38%)	8 (50%)	2 (11%)	4 (22%)	6 (33%)			

All *ns* between groups by.

*wks* weeks, *mo* month, *BG* basal ganglia, *PVWM* periventricular white matter, *ICH* intracerebral hemorrhage, *IVH* intraventricular hemorrhage, *SAH* subarachnoid hemorrhage, *SDH* subdural hemorrhage.

^a^Mann-Whitney U test.

^b^Fisher’s exact test.

^c^Chi-Square test.

### Hypothermia and NVD interventions

For both acute and recovery groups (NVD-A and NVD-R) enrolled in the NVD dose-finding trial, we administered N-acetylcysteine (NAC, either 25 or 40 mg/kg/dose) and 125(OH)_2_D (calcitriol, either 0.03 or 0.05 μg/kg/dose) intravenously within 6 h of birth and continuing for 10 days in neonates who qualified for 72 h of TH to 33–33.5 °C rectal temperature, as previously described.^12,13^ The control group (HIE-C) received 72 h of hypothermia only.

### Neurodevelopmental outcomes assessments

All cohorts had standard developmental testing in high-risk follow-up clinic, which included the Peabody Developmental Motor Scales to assess gross motor (GM) delays, the Cognitive Adaptive Test (CAT) and the Clinical Linguistics and Auditory Milestone Scale (CLAMS), with mean standard score 100 ± 15. Modified Checklist for Autism (MCHAT) screening was performed at 18–24 months of age.

### dMRI data collection

MRI scans were performed on unsedated infants, during natural sleep, after feeding and swaddling, using 3 T MRI scanners: Skyra [NVD-A], Trio [NVD-R] and Vida [HIE-C] (Siemens Healthineers, Erlangen, Germany). The MR protocol included T1- and T2-weighted, and MR spectroscopy scans. DKI scans were gathered with 3 *b*-values, *b* = 0, 1000 and 2000 s/mm^2^, TE = 99–141 ms, TR = 4800–6000 ms, voxel size = 12–27 mm^3^, and either 30 or 64 diffusion encoding gradient non-zero (Table 2). There were also 10 extra non-diffusion weighted images (*b* = 0) collected with matching image acquisition parameters. The DKI protocols were the same within a cohort, though scanners varied across cohorts as detailed in Table 2. In most cases, scans were completed within 60 min. All MRI scans were read by a single neuroradiologist.

**Table 2:** DKI protocols (*b*-values = 0, 1000 and 2000 s/mm^2^)

	3 T MRI	TE (ms)	TR (ms)	Voxel size (mm^3^)	Directions per *b*-value	Acquisition time
NVD-A	Skyra	109	6000	2 × 2 × 3	30	4 m
NVD-R	Trio	99	4800	(2.7)^3^	64	6 m 40s
HIE-C	Vida	141	6000	(3.0)^3^	64	14 m 12s

### Image processing

**Fig. 2:**
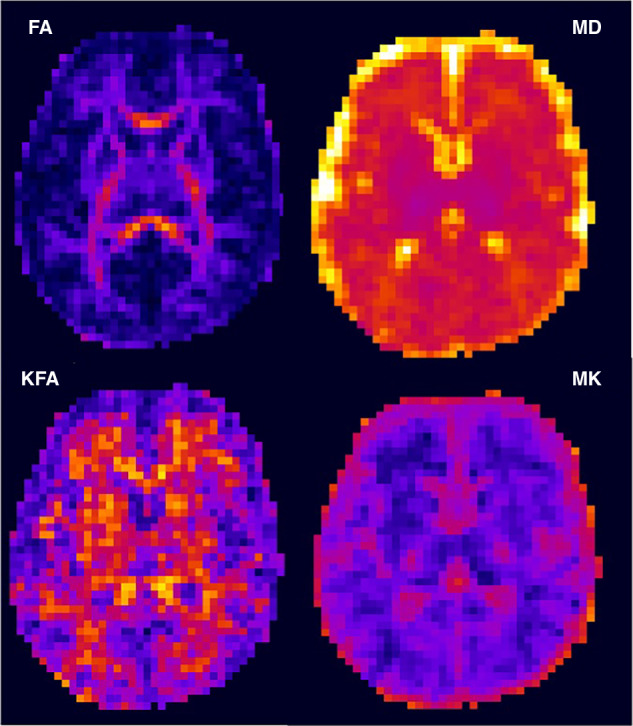
Diffusivity and kurtosis axial image from a 43-week 1-day GA neonate that had no pathologic findings on the MRI reading. Top row, conventional diffusivity measures of fractional anisotropy (FA) and mean diffusivity (MD). Bottom row, kurtosis fractional anisotropy (KFA) and mean kurtosis (MK). The diffusivity has units of μm^2^/ms. While the FA is quite low in most regions (deep purple) except the genu and the splenium of the corpus callosum, the KFA shows higher values (red/yellow) with more diversity in the reported higher-order anisotropy, reflecting tissue complexity. Within the MD map that the thalami (purple voxels) are approaching their asymptotic lowest value, while the MK map shows greater detail in this region. Interestingly, the MK has low values within the WM, with slightly higher values in the cortex and thalamic gray matter. Eventually, during myelination and maturation, the MK in the WM will exceed that in the gray matter due to the restriction from biological barriers, such as myelin.

**Fig. 3:**
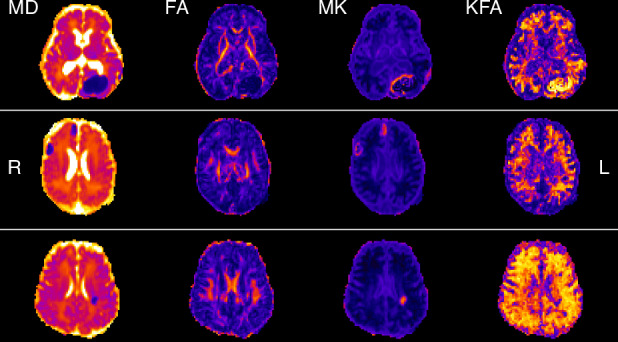
Example mean diffusivity (MD), fractional anisotropy (FA), mean kurtosis (MK) and kurtosis fractional anisotropy (KFA) images in three different subjects (rows) with various infarcts. Kurtosis provides complimentary information that supplements and does not duplicate diffusivity. On the top row, a 41-week GA neonate experienced a left occipital lobe hemorrhage that developed during ECMO. In the middle row, MRI in a 37-week GA neonate shows signal abnormalities in the ventrolateral thalami and posterior putamen, along with right frontal, parietal, temporal and cerebellar hemorrhages. The infant also had a right sided grade 2 IVH. Lastly, the neonate on the bottom row is 40 week GA with a left posterior parietal lobe hemorrhagic venous infarct. All three neonate images shown come from the NVD-A cohort where the infarcts were imaged within the first week of life. These images demonstrate a stark contrast between the diffusivity and kurtosis metrics. While the infarcts are represented as a void on the MD and FA, they are bright on the MK and KFA, illustrating the increased complexity within the infarct. This points to a potential use for MK and KFA as more sensitive markers of recovery dynamics within damaged brain tissue.

DKI data were quality controlled by removing image volumes and associated gradient directions containing excessive head motion or signal drop-out artifacts. Cleaned DKI data were processed using PyDesigner^42^ resulting in rotationally invariant parametric maps derived from the estimated diffusion and kurtosis tensors. Diffusion parameters calculated and used in our analysis included MD, FA, MK and KFA. Representative parametric maps in a single neonate are shown to highlight the differences in diffusivity and kurtosis metrics in Fig. 2. Example images of neonatal brain infarcts as viewed with both diffusivity and kurtosis are provided in Fig. 3.

### Region-of-interest analysis

**Fig. 4:**
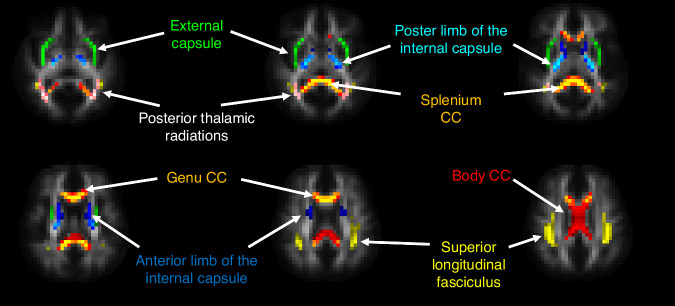
Regions of interest from the Johns Hopkins white matter atlas used in the analysis overlaid on a fractional anisotropy image.

For each cohort, each subject’s FA image was registered to the Johns Hopkins WM atlas. Transformation matrices were then inverted, and WM ROI were down sampled into the native diffusion space of each subject for analysis. Voxels were filtered within each ROI by applying an FA > 0.1 and WM mask probability at 50%. Remaining voxels were then averaged for each diffusion metric and for each ROI. The WM ROIs analyzed (Fig. 4) were the bilateral anterior and posterior limb of the internal capsule (ALIC/PLIC), the external capsule (EC), the corticospinal tract at the level of the cerebellar peduncle (CSTcp), the posterior-thalamic radiations (PTR), the superior longitudinal fasciculus (SLF), and the splenium, body, and genu of the corpus callosum (sCC/bCC/gCC).

### Area deprivation index (ADI)

We used the area deprivation index (ADI) to measure resource poor environments as a co-variate in neurodevelopmental outcomes. ADI is a standardized index that includes the household, education, employment, housing quality, and income measures taken from the US census and American Community Survey data.^43–45^ Scores range from 1 to 100% with higher percentiles indicating more disadvantaged areas based on national levels. ADI has been validated in several studies showing that neighborhood disadvantage is important to consider in child development and well-being.^43,45–47^ We referenced the ADI by national percentile for each infant according to home address to assesses the impact of the socioeconomic status on neurodevelopment.

### Statistical analysis

We only analyzed MRI data within cohort, to assess comparisons among infants scanned under the same imaging protocol, using SAS (SAS Institute Inc, Cary, NC). For each cohort, we first assessed if there were any group differences in the diffusion metrics based on encephalopathy stage using Wilcoxon rank sum test. Next, we determined if NAC dose was associated with differences in diffusion metrics. Then we used general linear modeling with stepwise selection of *p* ≤ 0.1 as inclusion criteria and *p* ≤ 0.05 to remain in the final model to evaluate the relationship of a diffusion metric across ROIs with the GM, cognitive and language outcome scores. Linear model selection in a stepwise model selection was done by maximizing adj-*R*^2^ values. All modeling included clinical variables of sex, NE stage, vitamin D dose, NAC dose, GA at birth and at MRI, and ADI as covariates. Due to the small sample size in each cohort and the exploratory nature of the study, multiple comparison correction were not performed.

## Results

### Demographics

For all groups, the average GA was 38–39 weeks at birth and infants were approximately equally divided between stage 2 and 3 HIE (Table 1). All diffusion sequences were obtained after rewarming when neonates had been normothermic for ≥ 24 h, at median (IQR) postnatal day: NVD-A 5 (5–6), NVD-R 23 (14–42), HIE-C 6 (5–7). Developmental testing scores are listed in Table 3, with the mean ages ± SD in months at developmental follow-up were 31 ± 15 (NVD-A), 34 ± 15 (NVD-R), and 17 ± 7 (HIE-C).

**Table 3:** Neurodevelopmental test scores

Gross motor		NVD-A	NVD-R	HIE-C	*p*^*a*^
Stage 2	Mean (SD)	101 (16)	103 (14)	87 (12)	0.55
	range	76–120	76–120	69–105	
	*N*	5	7	10	
Stage 3	Mean (SD)	94 (13)	92 (19)	96 (28)	0.92
	range	66–104	57–115	38–140	
	*N*	8	9	9	
	*p*^b^	0.28	0.24	0.19	

Cognitive adaptive test		NVD-A	NVD-R	HIE-C	*p*
Stage 2	Mean (SD)	96 (17)	97 (14)	85 (22)	0.37
	range	71–118	71–118	29–109	
	*N*	5	7	10	
Stage 3	Mean (SD)	88 (28)	85 (32)	92 (12)	0.85
	range	25–115	25–115	48–122	
	*N*	8	9	9	
	*p*^b^	0.82	0.60	0.55	

Clinical linguistics and auditory milestone scale		NVD-A	NVD-R	HIE-C	*p*^a^
Stage 2	Mean (SD)	91 (10)	95 (8)	92 (15)	0.84
	range	83–103	64–117	65–108	
	N	5	7	10	
Stage 3	Mean (SD)	86 (21)	81 (27)	104 (27)	0.15
	range	38–104	38–115	61–161	
	N	8	9	9	
	*p*^b^	1.00	0.33	0.34	

^a^ANOVA *p* value.

^b^Mann–Whitney U *p* value.

### DKI reflects clinical NE severity

**Fig. 5:**
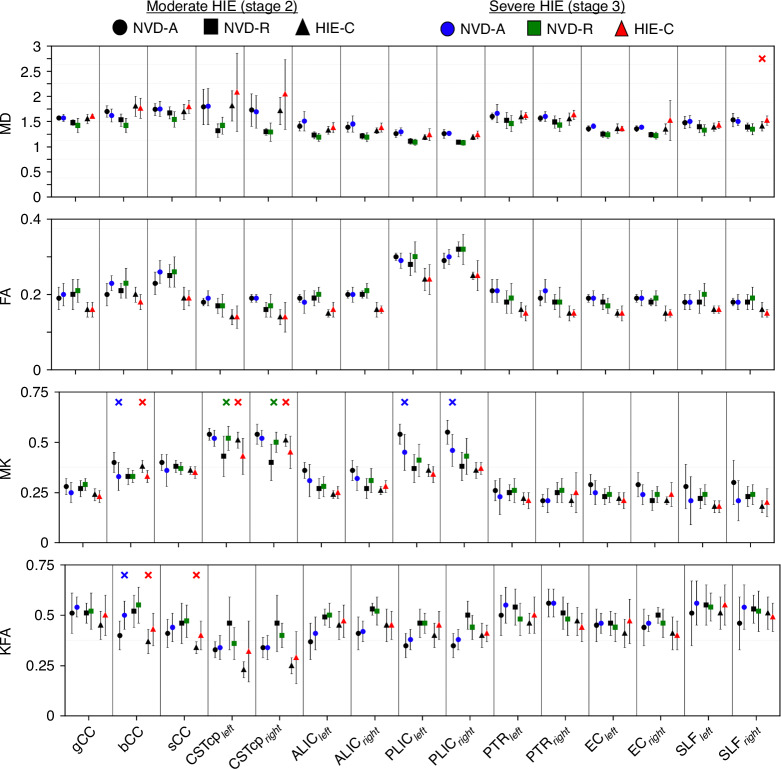
An overview of the average mean diffusivity (MD), fractional anisotropy (FA), mean kurtosis (MK) and kurtosis FA (KFA) separated by cohort and HIE stage for each white matter (WM) region in the anlaysis. These plots are only raw metric values with no adjusting for covariates performed. The colored “x” above specific WM regions for a given dMRI metric are those that were significantly different (*p* < 0.05), without correcting for multiple comparisons, between stage 2 and 3 HIE groups within a cohort. These match those reported in Table 4. NVD-A: blue circles; NVD-R: green square; and HIE-C: red triangle. Colored markers: stage 3 (severe HIE); Solid black markers: stage 2 (moderate HIE). All metrics are unitless except for MD with units of μm^2^/ms.

Table 4 presents the results for significant differences (*p* < 0.05) in diffusivity and kurtosis metrics within each NE severity group, specifically in the corresponding ROIs, along with the associated *p* values. Figure 5 plots the mean diffusion metric and standard deviations within each ROI for all NE groups, separated by severity. Those plots with significant differences between NE severity within cohort are marked with an “x” above them and correspond to the results in Table 4. Figure 5 plots show raw data, not adjusted for covariates or multiple comparisons correction.

**Table 4:** Comparison of diffusion metrics by HIE severity

	Metric	Stage 3 vs Stage 2	ROIs	p^*^
*NVD-A*	MK	Decreased	bCC	<0.01
		Decreased	Right/Left PLIC	<0.05 (both)
	KFA	Increased	bCC	<0.05
*NVD-R*	MK	Increased	Right/Left CSTcp	<0.05 (both)
*HIE-C*	MK	Decreased	Left CSTcp	<0.05
		Decreased	bCC	<0.01
		Decreased	Left PLIC	<0.05
	KFA	Increased	sCC	<0.05
		Increased	Left PLIC	<0.05
		Increased	Left EC	<0.05
	MD	Increased	Right SLF	<0.05

*p-value calculated using Wilcoxon rank sum test.

*bCC* bilateral corpus callosum, *sCC* splenium of corpus callosum, *CSTcp* corticospinal tract at the cerebellar peduncles, *EC* external capsule, *ALIC* anterior limb of the internal capsule, *PLIC* posterior limb of the internal capsule, *SLF* superior longitudinal fasciculus.

In the acute scans from both NVD-A and HIE C groups we found decreased MK in bCC and the left PLIC in stage 3 compared to stage 2 encephalopathy. (Table 4), indicative of greater injury to axonal structures. In the recovery period (the NVD-R group), the MK was increased in infants with stage 3 compared to stage 2 encephalopathy in the bilateral CSTcp, which may be consistent with repair and/or gliosis after HI inflammation.^31^ We also noted increased KFA in the acute severe stage of NE, indicating non-Gaussian movement of water with greater directional variation from the main fiber direction, such as might occur with edema or injury to the cytoskeletal or cellular membranes.^48,49^ The acute scans in the NVD-A group and HIE-C groups again had strong agreement in the findings of increased KFA in CC regions in stage 3 compared to stage 2 encephalopathy.

In considering NAC dose effect on WM microstructure, we found the FA in the right PTR (*p* < 0.05) and the left EC (*p* < 0.05) to be higher in convalescing scans from NVD-R subjects that received NAC 40 mcg/kg/dose (*n* = 6) than those who received 25 mcg/kg/dose (*n* = 12), perhaps indicating better preservation of cellular structures. MK in the acute scan was greater in the left SLF (*p* < 0.05) in the NVD-A subjects that received NAC 40 mcg/kg/dose (*n* = 3) compared to those that received 25 mcg/kg/dose (*n* = 13), although number of subjects was very small in this analysis.

### Long-term GM outcome modeling

We constructed generalized linear models for GM scores (Table 5) and adjusted for potential timing confounds, such as gestational age at birth and at the time of the MRI scan(s), by including them as model covariates in our NE cohorts. For the acute NVD scans, MD in the right PTR (adjusted for GA at MRI), and FA in the left PLIC (adjusted for vitamin D dose) estimated the GM score best (Table 5). For the recovery NVD-R scans, MK in the left CSTcp, ALIC, and PLIC (adjusted for GA at birth) estimated the GM scores well. Also, in the recovery NVD-R scans, FA in the left CSTcp, the gCC, the sCC, and the right PLIC, along with ADI and vitamin D dose, estimated the GM scores well. Importantly, the recovery scan FA and MK models explained more of the variability in GM scores (higher adjusted *R*^2^ value), than the acute scan model in the NVD group. Additionally, the KFA in the right PTR and bilateral SLF (adjusted for GA at MRI) also contributed to explaining a some of the variability in the GM scores.

**Table 5:** Linear models of GM scores

	Metric	Final model	p^*^	Adj-*R*^2^
NVD-A	MD	Right PTR, GA MRI	<0.005	0.65
	FA	Left PLIC, Vit-D dose	<0.01	0.58
NVD-R	MK	Left CSTcp, Left ALIC, Left SLF, GA birth	<0.0001	0.81
	KFA	Right PTR, Right/Left SLF, GA MRI	<0.001	0.81
	FA	Left CSTcp, gCC, sCC, Right PLIC, Vit-D dose, GA MRI, ADI	<0.001	0.95
HIE-C				
	KFA	CSTcp, right PLIC, left EC, GA MRI	<0.001	0.83
	MD	Right CSTcp, left PLIC, PTR, right EC, GA birth	<0.001	0.94

*p-value is the full final model with selection entry/stay *p*-value set to 0.1 and 0.05.

In the HIE-C group, FA in acute scans was not predictive of later GM scores. The MD (adjusted for GA at birth) and KFA (adjusted for GA at MRI) from the acute scans were the best metrics for estimating GM outcomes, particularly in the CSTcp, PLIC, and EC. Interestingly, NE clinical stage did not significantly contribute to diffusion models of outcome using the acute or recovery scan data in either NVD or control HIE groups.

### Long-term cognitive outcome modeling

Models were constructed for CAT (Table 6) and CLAMS (Table 7) cognitive outcomes in our HIE cohorts, adjusting for the same covariates as the GM models. Tables 6 and 7 provide full model details, including significant clinical covariates. Briefly, in the acute scans in the NVD-A and HIE-C groups, FA and MD metrics combined in various ROIs yielded the best predictive models for cognitive outcome by CAT scores (Table 6). In the recovery scan (NVD-R), FA in the right PLIC, and MK in the CC and bilateral PLIC yielded the best models.

**Table 6:** Linear models of CAT scores

	Metric	Final model	p^*^	Adj-*R*^2^
NVD-A	MD	sCC, right PTR, Vit-D dose	<0.005	0.73
	FA	Left PLIC, Left EC, Vit-D dose	<0.005	0.72
NVD-R	MK	gCC, Right/Left PLIC, Vit-D dose	<0.001	0.84
	KFA	bCC, GA birth and MRI, Vit-D dose	<0.001	0.81
	MD	Right ALIC, GA MRI, Vit-D dose, ADI	<0.001	0.79
	FA	Right PLIC, GA birth and MRI, Vit-D dose, ADI	<0.001	0.92
HIE-C				
	KFA	right ALIC and left SLF	<0.0005	0.62
	FA	gCC, left ALIC, left CSTcp	<0.0005	0.65

*p-value is the full final model with selection entry/stay *p*-value set to 0.1 and 0.05.

**Table 7:** Linear models of CLAM scores

	Metric	Final model	p^*^	Adj-*R*^2^
NVD-A				
	FA	Right ALIC	<0.05	0.34
NVD-R	MK	gCC, sCC, GA birth, Vit-D dose	<0.005	0.68
	FA	right CSTcp, GA birth, Vit-D dose	<0.005	0.54
HIE-C				
	MD	PLIC and right EC	<0.005	0.52

*p-value is the full final model with selection entry/stay *p*-value set to 0.1 and 0.05.

As in prediction of GM outcomes, the vitamin D dose was again a significant determinant in cognitive scores in both acute and recovery scans in the NVD groups. The high adjusted-*R*^2^ values for the models indicate that most of the variability in the outcomes are explained by the diffusion metrics in the WM tracts and the vitamin D dose.

None of the diffusion models based on the acute scans predicted CLAMs scores particularly well, with adjusted *R*^2^ values ≤ 0.52 (Table 7). Of all three cohorts, MK in the gCC and sCC in the NVD-R group estimated CLAM scores best along with GA at birth and vitamin D dose.

## Discussion

DKI may be advantageous, especially in neonatal brain injury, as it accounts for the non-Gaussian decay observed in water diffusion when it meets barriers to water movement in the underlying tissue microstructure. Kurtosis is sensitive to complexities due to crossing fibers and extra- and intra-cellular membrane barriers. This feature may be particularly useful in the absence of myelination, but it has not been widely studied in the neonatal population. Moreover, kurtosis has the potential to provide a better understanding of HIE injury and state of repair than diffusivity.

Deep gray matter regions, like the basal ganglia, are primary targets of HIE injury that are typically reported, along with WM injury in the PLIC.^50–52^ However, there are additional WM regions essential for information processing and executing voluntary motor and sensorimotor tasks (e.g., ALIC, superior and inferior longitudinal fasciculi, EC, mid-cingulate gyrus motor area, supplemental motor area, premotor area, and the cerebellum).^52^ In general, regions such as these are underrecognized in research on neonatal brain injury and the clinical interpretation of conventional MRI scans. Therefore, we chose to highlight WM injury in these infants undergoing developmental plasticity as they recover from HIE, precisely because it is difficult to parse the injury using conventional MRI (i.e., anatomical T1 and T2-weighted imaging). As we report, DKI metrics in major WM tracts do significantly impact the predictive outcomes in our HIE infant cohorts.

In this study, we investigated diffusivity and kurtosis changes in WM ROIs in three NE cohorts, which differed in treatment and in time after presumed HI injury, with the recovery scans obtained during the time of pseudo-normalization of MD.^17,53,54^ Compared with published normative values for FA and MK,^32^ we observe values that are similar or lower than expected. For instance, in the CC regions, the FA is lower in both the gCC and sCC, while the MK is lower in the gCC but similar to normal values in the sCC. Our data demonstrate the value of the kurtosis metrics, MK and KFA, in differentiating the clinical severity of HI injury at two different time points after injury. In contrast, FA did not differentiate severity by clinical encephalopathy staging in these cohorts. Across different scanners, kurtosis showed higher sensitivity to the clinical severity of HI injury in multiple WM tracts (Table 4). Also consistent across cohorts, we found that MK was lower acutely in severe (stage 3) compared to moderate NE subjects (stage 2) in the CC and the PLIC, while KFA was increased, consistent with more intracellular disruption of architecture. With FA, a strong directionality to the water movement frequently indicates greater intact axonal packing, bundling, and fiber coherence, whereas higher MK in acute scans indicates greater complexity of extra- and intracellular barriers, associated with less severe encephalopathy. Our data support the concept that diffusivity and kurtosis metrics measure different aspects of WM injury, recovery, and protection. Thus, dMRI in general provides novel data on specific CNS effects of treatment and dosing, which is unavailable from clinical factors.

The WM tracts used in these analyses are commonly affected in NE due to HIE and are key to determining long term neurodevelopmental outcomes.^55,56^ Another approach is to use a composite DTI score, an average of FA and MD from voxels in multiple anatomical regions. This has been used to relate to short term neurologic motor function of time to full oral feeding after HIE.^57^ For long-term outcome prediction, we regarded the specificity of WM tract microstructural injury/recovery as an important determinant of acute injury and functional recovery for different developmental tasks. Kurtosis and diffusivity metrics in these tracts from our recovery scans are central to our motor, cognitive, and language predictive models.

Neither the diffusivity or kurtosis metrics can definitively identify which specific cellular architecture or processes are disrupted by injury,^48^ but a recent hyperacute DKI study in neonatal piglets, showed that a smaller increase in MK as cytotoxic edema progressed over the first 24 h after HI injury, correlated with less mitochondrial membrane swelling and rupture, fewer autophagosomes and more normal mitochondria and was more sensitive to these cytoarchitectural changes than MD.^58^ Our data is largely obtained within 10 days after birth and after completing neuroprotective TH treatment in encephalopathic neonates. The decreased MK at this time point in our more severely encephalopathic neonates and in adults after stroke likely represents less complexity probed by water movement due to extensive disruption of cellular and axonal membranes, lower anisotropy in the axial direction, and more variability in the directionality of water movement.^30,59^

Besides sensitivity to injury severity, our data indicate that MK is sensitive to the scan timing after NE due to presumed HIE. MK was decreased in the acute scans after severe acute injury compared to moderate NE, indicating decreased complexity in WM ROIs. In later scans, MK was increased in severe NE infants. Our findings are supported by another study, which also found increased MK in the CC and cerebellar peduncles 2–3 months after HIE compared with scans obtained at 2–7 days.^60^ Thus, MK may also provide a window into cellular processes during recovery after severe and more moderate NE with a clinical diagnosis of HIE. Significant WM remodeling occurs during the recovery period after HI, with axonal sprouting, increasing crossing fibers, and axonal density, resulting in less organized, recovering WM tracts that may not be accurately captured by diffusivity.^30,61^ Our data is consistent with data in adult stroke that indicates diffusivity and kurtosis at different stages of recovery measure distinct aspects of WM injury and repair.

Ours and other data suggest that diffusivity and kurtosis metrics show response to therapeutic interventions, particularly in the recovery phase of NE, presumed due to HIE.^30,58,61^ Data from both acute and convalescing periods support an NVD treatment effect for stage 2 and 3 NE infants. NAC dose impacted both FA and MK in specific WM tracts, and vitamin D dose significantly contributed to models predicting GM and cognitive and language scores using either acute or recovery scans. The outcome modeling indicates that therapeutic and dose effects may be reflected in dMRI in these infants. In addition, differentiation of NE severity by kurtosis metrics in the acute phase of injury may have implications for therapeutics that target more severe injury.

To understand the pathophysiology of injury and recovery in infants as well as adults, which is essential for precision medicine, DKI provides novel kurtosis information reflecting tissue microstructural integrity that is not obtainable with diffusion tensor imaging. DKI has been applied in a variety of pathologies, such as stroke, epilepsy, Alzheimer’s disease, and Parkinson’s disease.^29,62–64^ For further testing of neuroprotection in cooled infants, independent validation of DKI in more severe injuries might help assess the response to treatment in different groups. Neurologic exams can and do change over the cooling period. Post-hoc determination of injury severity by DKI parameters after cooling would add another biomarker for the accurate assessment of whether specific treatments are valuable in the most severe NE group. DKI may also be helpful in prognostic determinations, providing more complete information to parents and determining eligibility for other early interventions.

DKI offers improved accuracy in estimation of diffusivity parameters, like FA and MD. However, it’s utility in neonatal brain injury is still being evaluated and warrants further study. We believe our and future DKI results can contribute to improved detection of injury and to patient care. Acquisition of DKI data is easily achieved on most modern clinical MRI systems by adding a *b*-value of 2000 s/mm^2^, with at least 15 directions, along with the lower *b*-value of 1000 s/mm^2^ with at least 6 directions to a typical DTI sequence. A DKI acquisition acquires 30 directions for each non-zero *b*-value with isotropic voxels, usually between (2.5 mm)^3^ and (3 mm)^3^. DKI also does not incur too great a time penalty, roughly between 5 and 8 minutes, depending on the scanner, which is crucial for its application in neonates. Analyzing DKI data is also straightforward for those familiar with image processing using currently available tools, which provide a standard pipeline of process steps to acquire and analyze these data. Furthermore, DKI and DTI metrics can be obtained from the same DKI scan.

Limitations of this study include using different scanners over time with different protocols. While this is a limitation, it is also a strength, as scanner and protocol upgrades are constantly occurring, and any dMRI derived metric will need to be robust despite these changes. Our findings do not depend on specific protocols or scanners, and yet show that kurtosis may offer prognostically important information in neonatal HIE. Additional limitations include a lack of multiple comparisons corrections due to the exploratory nature of the study, the small sample sizes, and the absence of an absolute gold standard for NE severity. All NE stage scores were determined at enrollment or admission to the NICU, as in any therapeutic trial design. Nevertheless, our data obtained at 5–9 days of life indicate that kurtosis can better capture and represent severity of acute encephalopathic injury after birth than diffusivity.

Another limitation is the lack of enrolling a normative control group, without any injury. While we could have used scans of neonates that were obtained for diagnostic purposes but read as “negative,” there is no assurance that the infants who have MRI scans for cause do not have some WM injury that is undiagnosed clinically or by conventional MRI. Normative values for diffusivity and kurtosis have been previously reported in infants, ranging in age from birth to approximately 4 years.^32^ For near-term infants, FA and MK both ranged between 0.2 and 0.6 in WM, with MK continuing to increase beyond two years of age, whereas FA plateaus. While it would be ideal to gather healthy neonatal DKI scans in the future, this was beyond the scope of the protocols and funding. Finally, our analysis focused only on DKI in WM tracts in predicting long-term outcomes, as these multiple tracts are primarily disregarded in published outcome models. The kurtosis metrics in deep gray matter regions (e.g., the basal ganglia) have been less well studied and may require significantly more work in both basic and clinical models for accurate interpretation of DKI changes.

## Conclusion

More accurate, quantitative neuroimaging is needed to fill the gap in accurate, early prognostication and provide better acute and chronic targeted interventions in early infancy. Our data indicate that kurtosis parameters, specifically MK and KFA, may offer complimentary information on CNS injury after NE presumed due to HIE, reflecting concurrent CNS injury severity, predicting the complexity of neurodevelopmental outcomes in conjunction with standard diffusivity measures, and demonstrating response to treatment in neonates treated for clinical HIE.

## Data Availability

In compliance with the IRB consents obtained during these periods of enrollment, the data are not publicly available but may be upon direct request to the author if it falls within the scope of the approved protocol and consent.
